# Beyond the first breath: comprehensive respiratory syncytial virus prevention through maternal immunization and infant immunoprophylaxis

**DOI:** 10.2478/abm-2025-0015

**Published:** 2025-06-30

**Authors:** Napaporn Chantasrisawad, Wicharn Boonjindasup, Thanyawee Puthanakit, Surasith Chaithongwongwatthana

**Affiliations:** Thai Red Cross Emerging Infectious Diseases Clinical Center, King Chulalongkorn Memorial Hospital, The Thai Red Cross Society, Bangkok 10330, Thailand; Center of Excellence for Pediatric Infectious Diseases and Vaccines, Faculty of Medicine, Chulalongkorn University, Bangkok 10330, Thailand; Division of Infectious Diseases, Department of Pediatrics, Faculty of Medicine, Chulalongkorn University, Bangkok 10330, Thailand; Division of Pulmonology and Critical Care, Department of Pediatrics, Faculty of Medicine, Chulalongkorn University, Bangkok 10330, Thailand; Department of Obstetrics and Gynecology, Faculty of Medicine, Chulalongkorn University, Bangkok 10330, Thailand

**Keywords:** bronchiolitis, infants, maternal immunization, monoclonal antibodies, passive immunization, respiratory syncytial virus

## Abstract

Respiratory syncytial virus (RSV) is a major respiratory pathogen that particularly affects infants under 6 months, premature infants, and those with congenital heart disease (CHD) or chronic lung disease. In 2019, there was estimated 3.6 million hospital admissions among children under 5 years of age due to RSV-related lower respiratory tract infection (RSV-LRTI), with more than 26,000 deaths. For decades, monthly palivizumab injection has provided passive immunization for high-risk infants and has demonstrated efficacy in reducing RSV-related hospitalizations, while breastfeeding has been known to protect against severe RSV-LRTI. Recent advances aiming to reduce severe RSV-LRTI, that is, bronchiolitis and pneumonia, include maternal RSV immunization and long-acting monoclonal antibodies for infants. Bivalent non-adjuvanted RSV vaccine (Abrysvo®), RSVPreF, administered during pregnancy (gestational age 24–36 weeks) transfers protective RSV IgG antibodies across the placenta with high cord-to-maternal ratio at ~1.5. Studies have shown that maternal immunization significantly reduced medically attended severe RSV-associated LRTI in infants, with an efficacy of 81.8% at 90 days and 69.4% at 180 days after birth, respectively. For medically attended RSV-associated LRTI, the efficacy was 57.1% at 90 days and 51.3% at 180 days. Additionally, long-acting RSV monoclonal antibodies (Nirsevimab) provide season-long protection with a single dose for infants during the first RSV season, reducing both medically attended RSV-LRTI and hospitalizations by approximately 70%–80% in infants during their first RSV season. Consequently, in 2024, the Strategic Advisory Group of Experts (SAGE) recommended that countries introduce maternal RSVPreF vaccination and/or RSV monoclonal antibodies for infant RSV prevention. Many countries have already adopted these interventions, demonstrating cost-effectiveness of monoclonal antibodies.

Respiratory syncytial virus (RSV) is one of the most significant respiratory pathogens affecting human populations worldwide, particularly vulnerable groups such as infants, young children, and those with chronic medical conditions, chronic lung disease, cardiovascular disease, or weakened immune systems. In young children, RSV is the most common cause of acute lower respiratory infection (LRTI), resulting in hospitalization and death [1, 2]. RSV is an enveloped, single-stranded RNA virus belonging to the order Mononegavirales, family *Pneumoviridae*, subfamily *Orthopneumovirus*, and species *Human orthopneumovirus* that causes infections of the respiratory tract ranging from mild cold-like symptoms to severe acute LRTI, including bronchiolitis and pneumonia [3]. The transmission of RSV occurs primarily through inhalation or direct contact with respiratory aerosols or secretions. This includes direct contact with respiratory droplets of infected people [3]. Mortality predominantly occurs in children with chronic medical conditions; in lower-middle incomes countries (LMICs), most mortality is seen in infants under 6 months of age [4]. This suggests that even in the absence of comorbidities, infants, particularly in resource-limited settings without intensive care, are at risk of dying from RSV infection due to their young age when the respiratory system and immune responses remain developmentally immature [4, 5].

This review will cover the epidemiology of RSV among children under 5 years, focusing on the Asia and Pacific region, and review the recent advances in RSV prevention strategies. The Strategic Advisory Group of Experts (SAGE) on immunization recommends that all countries should consider implementing passive immunization strategies such as maternal RSVPreF vaccination and/or monoclonal antibodies. The decision should consider cost, financing, supply, anticipated coverage, and feasibility within the existing health system [6].

## Epidemiology and seasonal pattern

### Burden of RSV in Asia-Pacific region

In 2019, there were estimated 33 million RSV-associated LRTI episodes globally in children under 5 years with 3.6 million hospital admissions [2]. Furthermore, approximately 26,300 children died from RSV-associated LRTI and 101,400 RSV-attributable deaths [2]. Young children, particularly infants under 6 months, and premature infants are at high risk for RSV [7]. Children with congenital heart disease (CHD), chronic lung disease, Down syndrome, neuromuscular disorders, and immunocompromised conditions also face an increased risk of severe RSV infection [8, 9].

In the Western Pacific and Southeast Asia regions, RSV accounted for 19% of all respiratory tract infections (RTI) and 29% LRTI in children under 5 years [10]. Several countries within the Asia-Pacific region have reported high RSV burdens. In Malaysia, RSV is the most commonly identified respiratory virus in children aged 6 months or younger with LRTI, constituting 81% of cases [10]. In Singapore, RSV is the most frequently detected virus among children hospitalized with RTI, with the highest positivity rates in those younger than 2 years. RSV accounted for 42% of bronchiolitis and pneumonia hospitalizations in children under 30 months in Singapore [1, 11]. China also faces a high burden of RSV infection, with a reported prevalence ranging from 17%–33% among children with severe acute respiratory illness [12]. Data from Thailand indicate that in the fiscal years 2015–2020, RSV-related LRTI accounted for 19,340 hospital admissions in children younger than 18 years [13]. In data from 2 Thai provinces between 2012 and 2014, RSV was identified in 20% of children under 5 years hospitalized with LRTI [14].

The economic burden of RSV in the Asia-Pacific region is also considerable. This health burden has a significant economic impact, with hospital care costs in LMICs ranging from US$243 to US$559, and in high-income countries from US$2,804 to US$7,037 [15]. In Thailand, the median total cost per RSV episode in children under 2 years of age was US$539, and US$2,112 for RSV with hospitalization [9]. Among hospitalized children in this Thai study, those with comorbidities had higher healthcare resource utilization and medical costs. The cost per hospitalization episode for RSV in Thailand was substantially higher than that of influenza (US$444 vs. US$2,112) [9]. In Singapore, the healthcare cost attributable to RSV infection is estimated at US$4.7 million annually [11]. These figures underscore the significant economic strain RSV places on healthcare systems and families in the region.

### Seasonal pattern of RSV in Asia-Pacific region

RSV is commonly circulated in months with cooler temperatures. The seasonality of RSV infection in the Asia-Pacific region exhibits considerable variation across different countries and climates; in some countries it is circulated year round or peaks in the rainy season [1, 12, 16]. This is contrasting with the more predictable winter seasonality observed in temperate regions [16,17,18]. Understanding the seasonality pattern of RSV in each country is indeed important data for RSV immunization implementation. Knowing these specific timings is crucial for healthcare providers and health officials to guide the administration of immunoprophylaxis to infants at high risk and to optimize the cost effectiveness of future vaccination programs by targeting the periods of highest RSV transmission. Data from Spain during the 2023–2024 winter season showed that implementing monoclonal antibodies for infants at birth in the month of November, the start of RSV season, cost €3,480 to prevent 1 RSV hospitalization, compared with a cost of up to €35,320 at the end of the season. This indicates that implementation later in the season results in higher cost per prevention [19].

In tropical regions, such as Singapore and Malaysia, RSV incidence can fluctuate throughout the year without distinct seasonal peaks [1, 17, 20]. However, studies in Malaysia have also shown a marked annual increase in RSV infections during November to January, which correlates with the number of rain days [21]. In Thailand, the peak admission rate for RSV-related LRTI occurs during the rainy season, from August to October [8, 13, 22]. This pattern of RSV activity aligning with the rainy season has also been reported in Vietnam, Indonesia, and the Philippines [1, 13]. By contrast, subtropical regions like Hong Kong and the northernmost area of Australia have RSV peaks occurring between the biannual influenza A virus peaks [1, 17]. Further north in Asia, Beijing, China, which experiences a temperate climate, typically sees annual RSV epidemics during the winter–spring months, from mid-October to mid-May [12, 23]. While the southern regions of China like Hong Kong have shown RSV seasonality in the spring–summer months [23]. This diverse landscape of RSV seasonality across the Asia-Pacific region highlights the influence of local climate factors, such as rainfall and temperature, and geographical location on the timing of RSV epidemics [1, 21]. Therefore, national and subnational surveillance data are essential to accurately determine the optimal start and duration for prevention and control measures in different geographic locations [18].

### RSV in pregnant and postpartum women

Pregnant women infected with RSV may be asymptomatic or present with a range of clinical manifestations of RTI. Most experience symptoms resembling the common cold, such as nasal congestion, cough, sore throat, and fever [24, 25]. However, some may develop signs of LRTI, such as shortness of breath and wheezing with duration of illness ranging from 7 days to 30 days [24].

Although severe cases are uncommon, complications such as pneumonia or worsening of preexisting respiratory conditions can occur and may require hospitalization [26, 27]. An analysis of data from over 1.6 million pregnant women between 2010 and 2016, conducted by the Pregnancy Influenza Vaccine Effectiveness Network, found that 2% of 846 women hospitalized with respiratory illness and tested for RSV were positive for the virus [27]. Of those RSV-positive cases, two-thirds occurred during the third trimester of pregnancy. Half of these women required hospitalization for 3 days or more, and 38% was diagnosed with pneumonia [27].

While direct fetal harm from RSV infection during pregnancy is not well established, maternal illness associated with RSV may increase the risk of preterm delivery [25, 27]. A meta-analysis of 5 studies, including 6,309 pregnancies and 33 RSV cases, did not find a statistically significant increase in the risk of preterm delivery [28]. Additionally, vertical transmission of RSV has been linked to pregnancy complications and adverse birth outcomes [29, 30], warranting further investigation to clarify its role in maternal and fetal health. In postpartum women, RSV infection poses additional concerns due to the potential transmission to newborns [31], who are at high risk for severe RSV-related illness. Maternal RSV infection may contribute to household transmission, emphasizing the importance of preventive measures such as hand hygiene, respiratory precautions, and vaccination strategies. While influenza virus infection poses increased risks of severe diseases and complications during pregnancy, RSV vaccination in pregnant women may not directly benefit the women themselves. However, it can offer protection to infants during their first 6 months of life, a concept similar to maternal pertussis immunization.

## Immunological consideration and transplacental antibody transfer

During RSV infection, both local and systemic immune responses, including cytokines produced by Th1 and Th2 responses, contribute to pathogenesis to varying degrees [32]. The inflammatory host immune response primarily causes airway damage, particularly in the bronchi and bronchioles. However, neutralizing antibodies generated later by the humoral immune response, in coordination with the Th1 response, play a crucial role in controlling viral replication and promoting clearance [32]. The serum neutralizing antibody and nasal IgA and IgG responses to the G (attachment) glycoprotein are specific to RSV A or B subgroups, whereas antibodies against the F (fusion) glycoprotein exhibit cross-reactivity between RSV groups [33]. During convalescence, circulating RSV IgG-producing memory B cells, but not IgA-producing ones, are present. This lack of mucosal IgA memory may contribute to repeated RSV infections, particularly in children [33, 34].

In adults who have previously been exposed to RSV, reinfection boosts serum antibody levels by reactivating memory B-cells, resulting in increased titers of anti-RSV IgG and IgA [34]. However, these elevated antibody levels are short-lived, typically returning to preinfection levels within 6 months [34]. Among antibody classes, IgG is unique in its ability to cross the placenta and provide passive immunity to the fetus. Of the IgG subclasses, IgG1 is transferred most efficiently, with reported cord-to-maternal blood ratios of approximately 1.5 [35]. RSV-specific antibodies are also efficiently transferred across the placenta; however, preterm infants exhibit significantly lower cord-to-maternal antibody transfer ratios compared with full-term infants [36]. A study of 291 Thai children found that 95% of newborns tested positive for anti-RSV IgG (≥16 relative units per milliliter), an antibody transferred from the mother via the placenta. However, these maternal antibody levels declined rapidly, with only 31% and 8% of infants remaining seropositive at 2 months and 7 months of age, respectively [37].

The mechanisms governing transplacental antibody transfer are complex; however, the neonatal Fc receptor (FcRn) plays a crucial role in mediating IgG transfer across placental syncytiotrophoblasts [35, 38]. The efficiency of this transfer depends on several factors including maternal IgG concentration, IgG subclass, glycosylation patterns, and FcRn binding affinity [35], as well as the expression levels of FcRn in placental tissue [38]. As pregnancy progresses and the placenta increases in cell mass, FcRn expression may also increase, leading to a higher rate of antibody transport [38]. These active transport mechanisms and protection from catabolism within the fetal circulation could contribute to higher apparent antibody levels in infants. This efficient transplacental transfer has been observed with antibodies against infections such as influenza and pertussis, where maternal immunization strategies have proven effective in reducing infant morbidity.

### Breastfeeding and RSV

Breast milk is rich in immunoglobulins, antimicrobial peptides, and immunomodulatory factors that help protect against a wide range of pathogens, including respiratory viruses [39]. Several studies have shown that breastfeeding is associated with significantly lower hospitalization rates, reduced oxygen requirements, and decreased mortality in infants with RTI [39,40,41,42,43].

A systematic review of studies conducted between 2000 and 2021, including 16,787 infants from 31 countries, found that both exclusive and partial breastfeeding reduced the severity of RSV-associated RTI, as well as hospitalization rates, length of hospital stay, need for supplemental oxygen, and intensive care unit (ICU) admissions [41].

In a prospective cohort study of 217 healthy infants followed from birth to 1 year of age, exclusive breastfeeding for the first 14 days of life provided notable protection against RSV hospitalization (odds ratio [OR] 0.21; 95% confidence interval [CI] 0.06–0.79) [42]. Among preterm infants born between 33 weeks and 35 weeks of gestation, breastfeeding for <2 months was identified as a significant risk factor for severe RSV infection requiring hospitalization (OR: 3.26; 95% CI: 1.96–5.42) [43]. Epidemiological studies across low-, middle-, and high-income settings have consistently demonstrated the protective benefits of breastfeeding in RSV prevention and supportive care, particularly for small, premature, and sick infants [42]. Breastfeeding should be actively promoted alongside vaccination in all public health communications and encouraged by healthcare providers during pre- and postnatal immunization visits as well as routine infant check-ups [44].

Exclusive breastfeeding reduces the severity and mortality of respiratory infection through several mechanisms, particularly benefiting small, premature, or sick infants. Breast milk provides a continuous supply of maternal antibodies, including IgA and IgG, which neutralize viruses in the infant’s respiratory tract and prevent viral attachment to the mucosal epithelium [39]. In addition to antibodies, human milk contains various protective factors, such as oligosaccharides, which inhibit pathogen adhesion to host cells [39]. These oligosaccharides may also reduce viral load and the inflammatory signaling in cultured RSV-infected human respiratory cells [45]. Furthermore, breast milk includes immune-modulating factors, such as cytokines and growth factors, that promote the development of the infant’s immune system and enhance its ability to combat infection [45]. In premature infants, breast milk is particularly crucial as it compensates for their immature immune systems and provides essential nutrients and growth factors that support lung development and reduce the risk of severe RSV disease [39, 42, 43].

## RSV infection in infants

### Pathophysiology of RSV-related lower respiratory tract infection

RSV attaches to respiratory epithelial cells via G glycoprotein and fuses with the cell membrane using F glycoprotein, allowing viral entry and replication. RSV infection leads to acute inflammation of the bronchioles and alveoli, causing increased swelling of epithelial cells and sub-epithelial layers. The inflammation signals the recruitment of large numbers of lymphocytes, eosinophils, and neutrophils to the respiratory tract to eliminate infected cells. This results in epithelial necrosis. The NS2 protein of the virus also induces epithelial slough from the respiratory epithelium, leading to the accumulation of debris in the bronchioles and alveoli [46, 47]. Additionally, the inflammation usually disrupts the structure and function of ciliary cells, leading to excessive and viscous mucus production. RSV induces basal cell proliferation and transformation into mucus-producing goblet cells, worsening airway obstruction [47]. Partial obstruction results in air trapping, whereas complete obstruction leads to atelectasis. These pathological changes impair pulmonary ventilation and gas exchange, causing hypoxemia, hypercapnia, and increased work of breathing, which manifest as respiratory distress or respiratory failure.

### Risk factors for severe RSV diseases in infants

**Figure 1.**
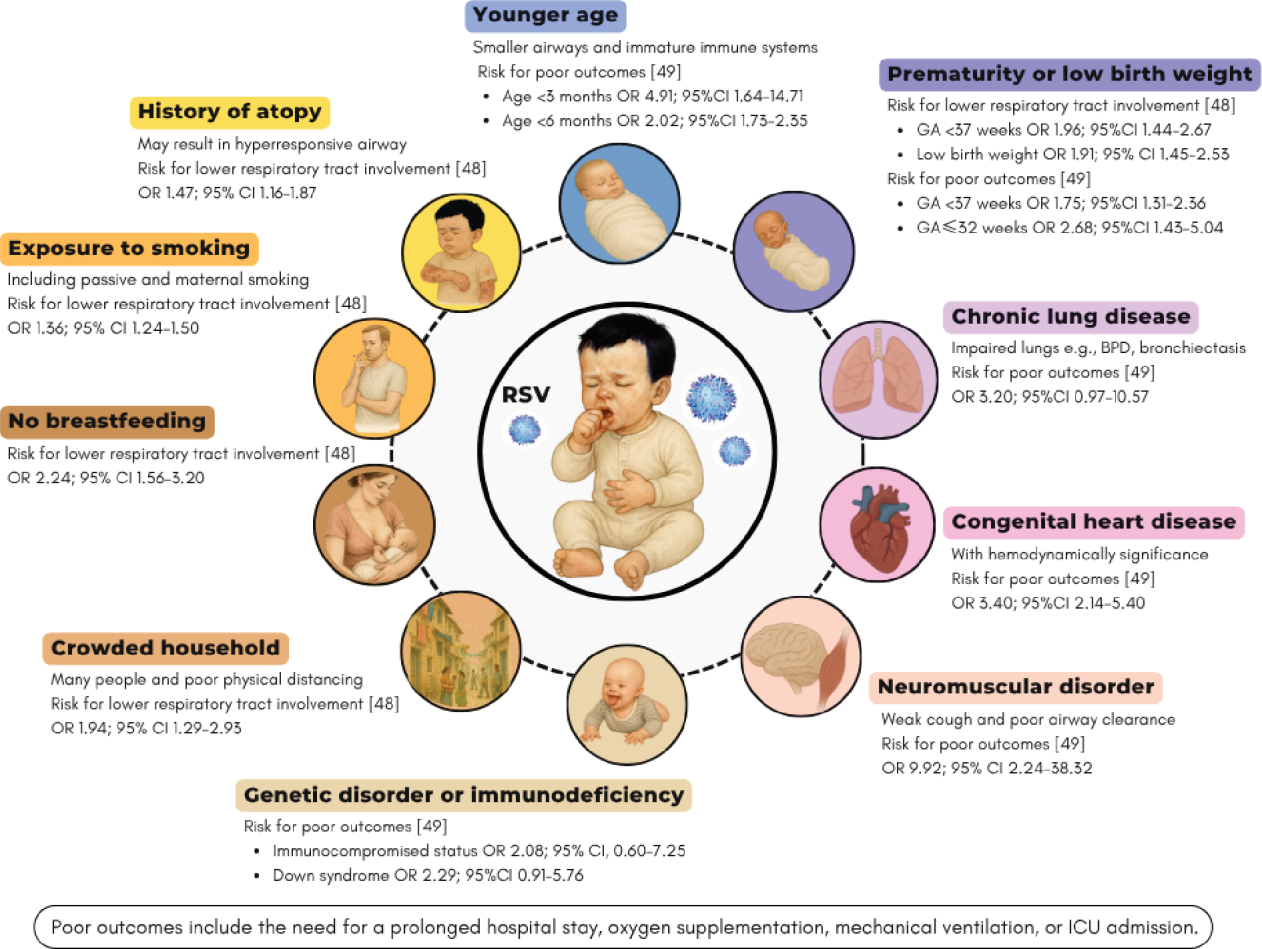
Risk factors for severe RSV infection and its impacts including lower respiratory tract involvement and poor outcomes. CI, confidence interval; ICU, intensive care unit; OR, odds ratio; RSV, respiratory syncytial virus.

Infants and children infected with RSV may develop more severe symptoms or a higher risk of mortality. The risk factors of severe RSV infection include child age, history of prematurity, and low birth weight [48]. Moreover, the presence of chronic underlying conditions such as CHD, chronic lung diseases, neuromuscular disorders, or genetic diseases places infants and children at higher risk of severe infection as any comorbid condition contributes to poor outcomes or death (OR: 2.69; 95% CI: 1.89–3.83) [49]. The severe symptoms during RSV infection usually result in prolonged hospitalization, prolonged oxygen therapy, respiratory failure requiring mechanical ventilation, or death. Additionally, environmental and family related factors have been associated with severe RSV infections. These factors include living in overcrowded households, lack of breastfeeding, and exposure to cigarette smoke or air pollutants [48, 49]. The impact of each risk factor is shown in **Figure 1**. These risks are often additive, meaning infants and children with multiple risk factors are at even higher risk of hospitalization and complications.

### Clinical manifestations of RSV in infants

In the early stages of RSV infection, patients typically exhibit upper respiratory symptoms resembling the common cold, including fever, cough, sneezing, rhinorrhea, lethargy, and poor feeding. In some cases, the infection proceeds to the lower respiratory tract, resulting in bronchiolitis or pneumonia. Symptoms of LRTI, such as dyspnea, tachypnea, progressive cough, wheezing, and crepitations or crackles, usually appear around day 3 after the onset of upper respiratory symptoms. Patients with significant LRTI often require hospitalization. Symptoms typically peak between days 3 and 5 before gradually improving, although some cases may deteriorate to respiratory failure [50].

Although extrapulmonary manifestations of RSV infection are rare, reports suggest that infants with RSV may experience seizures, hyponatremia, cardiac arrhythmias, heart failure, or hepatitis. Additionally, secondary bacterial infections may occur, prolonging illness duration [51].

### Additional investigations for RSV infections

Laboratory confirmation of RSV can be achieved through various methods. The commonly used diagnostic tests in clinical practice include rapid antigen tests and polymerase chain reaction assays using nasopharyngeal or oropharyngeal specimens. The rapid antigen tests to detect RSV have evolved over time, and been widely used in clinical settings, especially for pediatric populations. Routine testing is not always needed; however, it may impact clinical management or cohorting decisions. The sensitivity of rapid antigen tests is around 80%, though specificity is typically high (>90%). False negatives can occur, especially in patients with lower viral loads, later stages of illness, or specimens with inadequate sampling [52].

Although LRTI, including bronchiolitis and pneumonia, is clinically diagnosed, chest X-ray may be considered to assess respiratory involvement. It is important to note that the radiographic appearance of LRTI can vary in severity and there are no specific features for RSV. In viral bronchiolitis, common radiographic findings typically include hyperinflation, flattened diaphragms, patchy atelectasis, and peribronchial thickening. In viral pneumonia, common radiographic findings often include bilateral patchy or interstitial infiltrates, and may include signs of air trapping or atelectasis [53, 54].

### RSV severity scoring systems in infants and children

The assessment of RSV severity using a scoring system could assist in clinical assessment; screening for potential respiratory distress, predicting disease progression, guiding treatment planning, and monitoring patient status. A clinician can use a scoring system for RSV infection in various settings, including emergency departments, primary care, outpatient, and inpatient settings. Moreover, the scoring system is frequently used in research settings.

A systematic review of the literature published since January 2000 identified 31 severity scoring methods for children under 24 months with RSV infection and/or bronchiolitis [55]. The details of some RSV severity scoring systems are summarized in **Table 1**. Each scoring system incorporates key clinical parameters reported by clinicians to assess RSV infection severity, with variations in scoring methodology and interpretation.

**Table 1.** Comparison of RSV severity scoring systems in infants and children

**Scoring system**	**Assessment variables**	**Scoring method**	**Severity interpretation**
MTS [56]	- Respiratory rate - Wheezing or rales - Use of accessory muscles - Oxygen saturation (SpO_2_) in room air	Each variable scored 0–3	- No bronchiolitis: 0 - Mild: 1–5 - Moderate: 6–10 - Severe: 11–12
WBSS [57]	- General appearance - Respiratory rate - Wheezing - Chest retractions	Each variable scored 0–3, except general appearance (0–3)	- Mild: 0–3 - Moderate: 4–6 - Severe: 7–12
GRSS [58]	Age, respiratory rate, SpO_2_, retractions, wheezing, rales, cyanosis, nasal flaring, feeding difficulty	Continuous score from 0 to 10 based on logistic regression, requiring a software to calculate	Higher scores indicate more severe infection
PRESS [59]	- Respiratory rate - Wheezing - Use of accessory muscles - Oxygen saturation (SpO_2_) in room air - Feeding difficulties	Each variable scored 0 or 1	- Mild: 0–1 - Moderate: 2–3 - Severe: 4–5
RSV-CLASS [60]	- Cough - Tachypnea - Rales - Wheezing	Each variable scored 0 or 1	Higher scores indicate more severe infection

GRSS, global respiratory severity score; MTS, modified Tal score; PRESS, pediatric respiratory severity score; RSV, respiratory syncytial virus; RSV-CLASS, RSV clinical assessment severity score; WBSS, Wang bronchiolitis severity score.

Choosing the right RSV infection and/or bronchiolitis severity scoring system for real-world clinical use depends on many considerations, such as comprehensiveness, endorsement, validity, reliability, usability (simple and quick to apply), and responsiveness (the change in severity scores following disease progression or a treatment). Still, there is currently limited evidence to support that which score is clearly superior.

### Management of RSV-related lower respiratory tract infection

Currently, the treatment guidelines worldwide for RSV infection focus on respiratory care and symptomatic treatment. Children with mild upper respiratory tract symptoms that can be adequately managed at home may be treated as outpatients. However, hospitalization is strongly recommended for those with LRTI, risk factors for severe disease, or inadequate home care conditions. Hospital-based management should follow standard clinical guidelines for bronchiolitis and viral pneumonia in children, including fluid replacement, gentle suctioning and secretion clearance, oxygen therapy, and aerosol therapy as indicated [61, 62]. These interventions aim to alleviate symptoms, maintain adequate oxygenation, and support recovery from RSV infection. Oxygen should be provided when there is hypoxemia or any sign of respiratory distress. Some patients with severe symptoms may require heated humidified high-flow nasal cannula (HHHFNC). The effect of HHHFNC on avoiding treatment failure appears to be clinically meaningful for infants with acute bronchiolitis, including but not limited to RSV infection. The infants receiving HHHFNC remained free from treatment failure 7%–11% higher than those receiving standard oxygen therapy, with 46% risk reduction in emergency or early inpatient settings (relative risk [RR] 0.54; 95% CI: 0.39–0.75) [63, 64].

The only currently approved antiviral agent for RSV infection is Ribavirin. Ribavirin has been available for many decades in aerosol and oral forms. A recent meta-analysis has revealed no differences in mortality in patients with RSV infection treated with aerosol/oral ribavirin compared with supportive care (RR: 0.63; 95% CI: 0.28–1.42), but significantly lower mortality in the subgroup of hematological patients (RR: 0.32; 95% CI: 0.14–0.71) [65]. Still, Ribavirin is not recommended for RSV infection in most countries due to its limited efficacy and potential adverse reactions, such as anemia, liver toxicity, and respiratory irritation from its aerosolized particles [65].

Novel efficient and affordable antiviral agents for RSV are urgently needed. The antiviral agents currently in clinical development for RSV infection include drugs that inhibit the entry process of RSV, that is, attachment or fusion inhibitors, and drugs that target RSV after entry, for example, replication inhibitors [66]. Among the existing candidates, Ziresovir appears to be the most probable to achieve real-world clinical use in pediatrics, based on its efficacy in reducing clinical score and RSV viral load, and its safety compared with placebo as demonstrated in phase 3 clinical trials [67, 68].

### Potential long-term consequences

LRTI caused by RSV can have long-term effects on the respiratory system, especially the infection during infancy. Following an RSV infection, wheezing illnesses have been reported in 30%–50% of cases during the first year, then gradually decreasing to 20%–30% by the third year [69]. A recent large cohort study enrolling almost 1,800 children found that the prevalence of asthma at the age of 5 was significantly higher among children who had experienced an RSV-related lower respiratory tract infection (RSV-LRTI) in the first year of life compared with those who had not (21% vs. 16%, respectively). Avoiding RSV infection during this early period was associated with a significantly lower risk of developing asthma (adjusted RR: 0.74; 95% CI: 0.58–0.94) [70]. A meta-analysis of 15 studies, encompassing data from approximately 82,000 children of whom 1,533 with RSV-confirmed found that hospitalization due to RSV infection in early life was associated with a significantly increased risk of developing wheezing or asthma later in childhood (OR: 3.84; 95% CI: 3.23–4.58). The heterogeneity among the included studies was low to moderate, indicating consistent findings across different populations [71]. Therefore, the perspective of cost analysis for RSV prevention should account for RSV-associated burden of diseases, not only acute LRTI, but also long-term consequences on the risk of childhood wheezing or asthma [72,73,74].

## Recent advance in RSV prevention strategies using passive immunization

Developing an effective RSV vaccine for infants is challenging because the disease is most severe in the first 6 months of life, a period when their immune systems are still immature and vaccination may not provide timely, complete protection. There was no current approved RSV vaccine for infants and children. Initial attempts at vaccination in the 1960s utilizing formalin-inactivated RSV vaccine demonstrated not only inefficacy but precipitated enhanced respiratory disease upon natural infection [75]. More recently, mRNA vaccines were explored; however, a Moderna trial in infants, aged 5–24 months, was paused due to more cases of severe RSV illness in vaccine groups, raising concerns for vaccine-associated enhanced respiratory disease (VAERD). Consequently, Moderna discontinued its infant RSV mRNA vaccine programs [76]. Current efforts focus on live-attenuated intranasal vaccines like RSV/6120/ΔNS2/1030s and RSV/ΔNS2/Δ1313/I1314L, which aim for better safety and immunogenicity [77].

Contemporary prophylactic strategies emphasize passive immunization via maternal immunization and monoclonal antibodies for infants. Transplacental antibody transfer during gestation provides neonates with immediate though transient protection, with efficacy contingent upon gestational duration and maternal antibody titers [75]. Direct administration of RSV-specific antibodies represents the second immunoprophylactic approach.

### RSV vaccines and maternal immunization strategies

Maternal immunization aims to provide protection for newborns during the first 6 months of life through the transplacental transfer of vaccine-induced antibodies. Maternal immunization against tetanus, influenza, and pertussis is well-established as an effective strategy to prevent these infections in infants. This approach has also been applied to RSV vaccination in pregnant women, aiming to reduce the risk of severe RSV-related illness in newborns. The bivalent RSVPreF (Abrysvo®), a recombinant subunit RSV vaccine without an adjuvant, is currently the only RSV vaccine approved for use in pregnant women to protect newborns from RSV infection. It received approval from both the United States Food and Drug Administration (USFDA) and the European Medicines Agency (EMA) in 2023. The USFDA approved its use between 32 weeks and 36 weeks of gestation, while the EMA authorized its use between 24 weeks and 36 weeks [78]. In Thailand, the national regulatory agency approved the vaccine in July 2024, for use during 24–36 weeks of pregnancy.

The MATISSE study was a landmark study, phase 3 randomized clinical trial conducted in pregnant women to evaluate the safety and efficacy of the bivalent RSVPreF vaccine (120 μg) compared with a placebo [79]. A single intramuscular injection of the vaccine or placebo was administered between 24 weeks and 36 weeks of gestation. A total of 3,682 women received the vaccine and 3,676 received the placebo. The women were then monitored for adverse events and safety outcomes for 6 months postpartum for mothers, and their infants were followed for 12–24 months after birth. The study found that most vaccine-related reactions in pregnant women were mild to moderate, with local side effects occurring more frequently among the vaccinated group. Pain at the injection site was the most common side effect. However, the proportion of mothers experiencing adverse events within the first month post-vaccination was not significantly different between the vaccine and placebo groups (13.8% vs. 13.1%). Similarly, there was no difference in the incidence of serious maternal adverse events up to 6 months postpartum, including preterm birth, between the 2 groups. No serious adverse events in infants monitored from birth through 24 months were considered related to the vaccine [79]. The study showed maternal immunization significantly reduced medically attended severe RSV-associated LRTI in infants, with an efficacy of 81.8% at 90 days and 69.4% at 180 days after birth, respectively. For medically attended RSV-associated LRTI, efficacy was 57.1% at 90 days and 51.3% at 180 days [79] (**Table 2**). Findings from the MATISSE study supported approval of the bivalent RSVPreF vaccine for use in pregnant women to protect newborns against RSV infection by various national regulatory agencies. In addition, the World Health Organization (WHO) and other organizations have issued recommendations on its use. However, these guidelines differ regarding the recommended gestational age for administration and whether the vaccine should be given seasonally or year-round [6, 78, 80,81,82] (**Table 3**).

**Table 2.** Vaccine efficacy of bivalent RSVPreF during pregnancy to prevent LRTI in infants from the MATISSE study [79]

**Outcomes**	**Vaccine efficacy % (95% or 97.58% CI)**
Medically attended severe RSV-associated lower respiratory tract illness in infants	
• 90 days after birth	81.8 (40.6–96.3)
• 120 days after birth	73.9 (45.6–88.8)
• 150 days after birth	70.9 (44.5–85.9)
• 180 days after birth	69.4 (44.3–84.1)
Medically attended RSV-associated lower respiratory tract illness in infants	
• 90 days after birth	57.1 (14.7–79.8)
• 120 days after birth	56.8 (31.2–73.5)
• 150 days after birth	52.5 (28.7–68.9)
• 180 days after birth	51.3 (29.4–66.8)

CI, confidence interval; LRTI, lower respiratory infection; RSV, respiratory syncytial virus.

**Table 3.** Recommendations for administering the bivalent RSVPreF vaccine in pregnant women in different countries [6, 78, 80,81,82]

**Country/organization**	**Gestational age (weeks)**	**Vaccination period**
WHO	≥28	Year-round
United Kingdom	≥28	Year-round
IDAT	24–36	Year-round
Austria	24–36	For births between September and March
Belgium	28–36	For births between September and March
Australia	28–36	Year-round
United States	32–36	September to January
Canada	32–36	Before late autumn to early spring
France	32–36	September to January
Argentina	32–36	March to August

IDAT, infectious disease association of Thailand; WHO, World Health Organization.

In March 2024, Argentina initiated a national maternal immunization program, employing bivalent RSVpref as the primary strategy to prevent RSV disease among infants. Pregnant women between 32 weeks and 36 weeks of gestation were offered the RSVpreF vaccine. The BERNI study, a multicenter, retrospective, test-negative, case–control study conducted across 12 Argentinian hospitals during the 2024 RSV season [83], evaluated the real-world effectiveness of RSVpreF vaccination during pregnancy in preventing RSV-associated lower respiratory tract disease (LRTD) leading to infant hospitalization during the first RSV season after implementation. The results showed high RSVpreF effectiveness against RSV-associated LRTD resulting in hospitalization for infants aged 0–3 months (78.6%, 95% CI: 62.1–87.9) and 0–6 months (71.3%, 95% CI: 53.3–82.3). Furthermore, the vaccine demonstrated 76.9% effectiveness (95% CI: 45.0–90.3) against RSV-associated severe LRTD leading to hospitalization in infants up to 6 months old.

### Recommendation of use of RSV maternal immunization in Thailand

The Infectious Disease Association of Thailand (IDAT), in collaboration with academic organizations, including the Royal Thai College of Obstetricians and Gynecologists (RTCOG), recommended the bivalent RSVPreF vaccine for pregnant women between 24 weeks and 36 weeks of gestation to prevent RSV-associated LRTI in infants from birth to 6 months of age. Administration between 28 weeks and 32 weeks of gestation is preferred to maximize protective benefit. Additionally, vaccination is strongly recommended when the infant is expected to be younger than 6 months during Thailand’s peak RSV season, which occurs from July to November [80].

In addition to the RSV vaccine, several other vaccines are routinely recommended for pregnant women in Thailand to protect both maternal and infant health. These are summarized in **Table 4** [80].

**Table 4.** Currently recommended vaccines for Thai pregnant women [80]

**Vaccine**	**Dosage and timing**
Td toxoid	0–3 doses, any trimester
Influenza vaccine	1 dose, any trimester (prefer 12–20 weeks)
Acellular pertussis vaccine	1 dose, ≥16 weeks (prefer 20–32 weeks)
COVID-19 vaccine	1 dose, any trimester (prefer 12–20 weeks)
RSV vaccine	1 dose, 24–36 weeks (prefer 28–32 weeks)

RSV, respiratory syncytial virus.

### Infant monoclonal antibodies strategies

The F glycoprotein of RSV stands out as a critical target for monoclonal antibodies due to its essential role in viral pathogenesis. This protein mediates membrane fusion between viral and host cellular membranes, enabling cell-to-cell viral spread and inducing characteristic syncytia formation [3]. Approved monoclonal antibodies target the F protein’s “Pre-F” conformational changes to effectively neutralize viral entry, blocking both initial infection and subsequent transmission by disrupting the virus’s fundamental mechanism of cellular invasion [84,85,86,87].

The first generation of monoclonal RSV antibody, palivizumab, has demonstrated safety and efficacy in high-risk infants for over 2 decades, despite limitations regarding cost and monthly administration requirements [75, 88, 89]. The second-generation monoclonal antibodies like nirsevimab and clesrovimab utilize a YTE (tyrosine, threonine, and glutamate) modification in their fragment crystallizable (Fc) receptor. This Fc modification extends their half-life, exemplified by nirsevimab’s 71–90 day half-life, providing season-long protection with a single dose [89,90,91,92]. These modifications enhance antibody stability and reduce clearance from the body, maintaining sustained levels of RSV-neutralizing activity after a single administration—improving convenience and potentially broadening coverage compared with earlier monoclonal antibodies requiring monthly dosing [86, 87, 91, 93, 94].

Regarding immunogenicity and resistance, nirsevimab has demonstrated low rates of antidrug antibodies (ADA) with no impact on efficacy or safety. Additionally, RSV exhibits low polymorphism at the nirsevimab binding site, with most viral isolates successfully neutralized [86, 87, 91, 93, 94]. Clesrovimab, while showing a higher incidence of ADA development (36.7%), did not experience altered pharmacokinetics in its phase 1b/2a trial. It targets a highly conserved epitope on the RSV F protein, suggesting minimal likelihood of resistance due to viral polymorphisms [92].

### Clinical implications and therapeutic strategies

Monoclonal antibodies like palivizumab and nirsevimab are crucial therapeutic strategies for preventing severe RSV, particularly in vulnerable infant populations [95]. Palivizumab, a humanized monoclonal antibody targeting the F protein of RSV, has been used for years, administered monthly via intramuscular injection for up to 5 doses during the RSV season [84, 85, 95, 96]. Its use is generally recommended for high-risk infants, including premature infants, children with bronchopulmonary dysplasia (BPD), and those with hemodynamically significant CHD [96]. More recently approved, nirsevimab is a long-acting monoclonal antibody that also targets the RSV F protein but offers the advantage of a single intramuscular dose providing protection for the entire RSV season in a broader infant population, including healthy preterm and term infants [87]. Both palivizumab and nirsevimab have demonstrated favorable safety profiles in clinical trials and real-world use [84,85,86, 91, 96]. Common adverse events associated with palivizumab include injection site reactions, fever, and diarrhea [96], while nirsevimab trials reported few drug-related adverse events [86]. Dosage for palivizumab is typically 15 mg/kg per dose [84, 85], while nirsevimab is administered as a single fixed dose (50 mg for infants weighing less than 5 kg and 100 mg for those weighing 5 kg or more) [87, 91] (**Table 5**).

**Table 5.** RSV monoclonal antibody comparison [84,85,86,87, 91, 92, 96, 104]

**Feature**	**Palivizumab**	**Nirsevimab**	**Clesrovimab**
Target site	Antigenic site II on the F protein	Antigenic site Ø on the prefusion F protein	Antigenic site IV of the F protein
Administration	Monthly intramuscular injection during RSV season (up to 5 doses)	Single intramuscular dose	Single intramuscular dose
Target population	High-risk (premature birth, BPD, CHD) infants and young children in subsequent seasons	All infants in their first season and high-risk children in subsequent seasons	All preterm and full-term infants in their first season, studied between 2 weeks and 8 months of age
Duration of protection	Monthly injections provide protection during the RSV season	Single injection provides protection for approximately 5 months	Extended half-life suggests potential for once-per-RSV season dosing
Efficacy	Reduces RSV hospitalization by 78% in premature infants, 39% in children with BPD, and 45% in children with CHD	Reduces RSV hospitalization by 62% in late premature and term infants, and 78% in premature infants (GA 29–35 weeks)	Reduces RSV-associated hospitalizations by 84% in infants
Safety profile	Generally safe, with similar adverse events to placebo. Some studies report slightly higher rates of fever and injection site reactions	Comparable to palivizumab, with no significant safety concerns	Generally well tolerated, with no treatment-related serious adverse events
Dosage	15 mg/kg of body weight per dose	Single dose of 50 mg (for infants <5 kg) or 100 mg (for infants ≥5 kg) and 200 mg in subsequent season	Single dose of 105 mg for infants
Engineering for long-acting	Mean half-life of 20 days following intramuscular administration	Extended half-life of approximately 69 days	Extended half-life of approximately 45 days

BPD, bronchopulmonary dysplasia or chronic lung disease of prematurity; CHD, hemodynamically significant congenital heart disease; RSV, respiratory syncytial virus.

Both monoclonal antibodies have shown significant efficacy in preventing RSV-related LRTI. Clinical trials of palivizumab in high-risk infants demonstrated a 55% reduction in RSV-related hospitalizations [84, 97, 98] and was associated with fewer RSV hospital days, reduced need for supplemental oxygen, and lower rates of ICU admissions [84]. Real-world studies have largely corroborated these findings, confirming palivizumab’s effectiveness in reducing RSV hospitalization rates in clinical practice [96, 97]. Nirsevimab has demonstrated even higher efficacy with a single dose, showing around 70%–80% reduction in medically attended RSV-associated LRTI and hospitalizations in healthy preterm and term infants during their first RSV season across multiple phase 2 and 3 trials [86, 87, 99, 100]. Post-licensure, real-world studies across different countries, including France, Spain, and the United States, have confirmed nirsevimab’s substantial effectiveness in reducing hospitalizations due to RSV-associated bronchiolitis, with effectiveness estimates ranging from approximately 65%to 84% [99,100,101,102]. Network meta-analyses comparing different monoclonal antibodies indicate that both palivizumab and nirsevimab significantly reduce RSV-related infections and hospitalizations compared with placebo [103]. The extended half-life and single-dose administration of nirsevimab offer a significant advantage over the monthly regimen of palivizumab, potentially leading to broader protection and improved compliance [87, 91].

In June, 2025, USFDA approved clesrovimab in neonates and infants during their first RSV season. Clesrovimab administered as a fixed 105 mg dose, irrespective of weight, offering rapid and durable protection for a typical 5-month RSV season. This approval was largely based on the pivotal Phase 2b/3 CLEVER trial, which reported a 60.5% reduction in RSV-associated medically attended LRTI and an 84.3% reduction in RSV-associated hospitalizations over 5 months vs. placebo, with efficacy increasing with disease severity. The Phase 3 SMART trial further supported the approval, evaluating its safety and efficacy in infants at increased risk for severe RSV disease, where its safety profile was comparable to palivizumab. Clesrovimab has been recommend from the US Advisory Committee on Immunization Practices as an option for the prevention of RSV in infants younger than 8 months of age in their first season [104].

### Implementation and economic considerations

Several studies have examined the cost-effectiveness of nirsevimab compared with palivizumab for RSV prophylaxis in infants across several countries. In the United States, research on preterm infants (29 0/7–34 6/7 weeks’ gestational age) without additional risk factors found that palivizumab was not cost-effective compared with no prophylaxis. A threshold analysis based on a Phase 2b randomized controlled trial suggested nirsevimab could be cost-effective in the United States at prices below US$1,962 from a societal perspective [74]. In Canada, models indicated that replacing palivizumab with nirsevimab for high-risk infants could generate nationwide cost savings, though expanding nirsevimaab coverage to all infants would depend on pricing and regional factors [105]. A review in Canada noted that nirsevimab’s cost-effectiveness varied based on region, efficacy data, pricing, and RSV season severity [106]. Evidence suggests that nirsevimab could be a more cost-effective alternative to palivizumab, particularly given its single-dose administration compared with palivizumab’s multiple dose [74]. However, cost-effectiveness for both medications varies depending on the specific infant population, region, and healthcare system [74].

Replacing palivizumab with nirsevimab for high-risk infants appears cost-saving or cost-effective in several settings, but expansion of coverage and choice between different strategies (e.g., universal vs. targeted, seasonal vs. year-round) requires detailed local economic analyses. For nirsevimab, SAGE recommends a single dose using either a year-round approach (at birth or earliest opportunity) or a seasonal approach (for infants born during the RSV season or those ≤12 months entering the season). The seasonal approach may be more cost-effective in settings with clear RSV seasonality [6].

Both maternal RSVPreF vaccination and infant monoclonal antibodies offer effective strategies for preventing RSV in infants, with the optimal choice depending on a country’s specific context, including cost, supply, and healthcare infrastructure. Maternal vaccination leverages existing antenatal care, transferring protective antibodies to newborns via the placenta, but requires precise timing during pregnancy and alignment with local RSV seasonality. Conversely, newer, extended-half-life monoclonal antibodies like nirsevimab and clesrovimab provide immediate, season-long protection with a single infant dose, simplifying administration and expanding the target population to include healthy infants. However, their cost-effectiveness varies significantly, necessitating detailed local economic analyses, and effective implementation also hinges on understanding and timing with local RSV seasonality. Ultimately, deciding between these interventions, or combining them, requires a comprehensive assessment of local epidemiology, financial resources, supply chain capabilities, and healthcare system capacity.

## Conclusion

RSV presents a global health burden, particularly for vulnerable infants, leading to millions of hospital admissions and thousands of deaths globally, predominantly among infants under 6 months in lower-middle income countries. RSV infection in infancy is also linked to long-term consequences like an increased risk of developing recurrent wheezing or asthma later in childhood. Despite this substantial impact, there is currently no specific antiviral treatment widely recommended for RSV due to limited effectiveness and potential adverse effects; management primarily relies on supportive respiratory and symptomatic care. Consequently, recent efforts and advancements have focused on preventing severe disease through passive immunization strategies. Key prevention choices include maternal immunization with vaccines like bivalent RSVPreF administered during pregnancy, which transfers protective antibodies to the infant, and the use of long-acting monoclonal antibodies such as nirsevimab, providing season-long protection with a single dose. Both strategies have demonstrated high efficacy in reducing severe RSV-associated LRTI and hospitalizations in infants, leading expert groups to recommend their implementation to mitigate the burden of RSV in infants.
